# Human-animal interactions and machine-animal interactions in animals under human care: A summary of stakeholder and researcher perceptions and future directions

**DOI:** 10.1017/awf.2024.23

**Published:** 2024-05-09

**Authors:** Ellen Williams, Jennifer Sadler, Steven Mark Rutter, Clara Mancini, Christian Nawroth, Joseph M Neary, Samantha J Ward, Gemma Charlton, Annabelle Beaver

**Affiliations:** 1 Department of Animal Health, Behaviour & Welfare, Harper Adams University, Edgmond, Newport, UK; 2 School of Computing and Communications, The Open University, Milton Keynes, UK; 3 Research Institute for Farm Animal Biology, Dummerstorf, Germany; 4 Department of Livestock and One Health, Institute of Infection, Veterinary and Ecological Sciences, University of Liverpool, Liverpool, UK; 5 Animal, Rural & Environmental Sciences, Nottingham Trent University, Southwell, Nottinghamshire, UK

**Keywords:** animal-centred perspective, animals under human care, animal welfare, cross-industry collaboration, human-animal interactions, machine-animal interactions

## Abstract

Animals under human care are exposed to a potentially large range of both familiar and unfamiliar humans. Human-animal interactions vary across settings, and individuals, with the nature of the interaction being affected by a suite of different intrinsic and extrinsic factors. These interactions can be described as positive, negative or neutral. Across some industries, there has been a move towards the development of technologies to support or replace human interactions with animals. Whilst this has many benefits, there can also be challenges associated with increased technology use. A day-long Animal Welfare Research Network workshop was hosted at Harper Adams University, UK, with the aim of bringing together stakeholders and researchers (n = 38) from the companion, farm and zoo animal fields, to discuss benefits, challenges and limitations of human-animal interactions and machine-animal interactions for animals under human care and create a list of future research priorities. The workshop consisted of four talks from experts within these areas, followed by break-out room discussions. This work is the outcome of that workshop. The key recommendations are that approaches to advancing the scientific discipline of machine-animal interactions in animals under human care should focus on: (1) interdisciplinary collaboration; (2) development of validated methods; (3) incorporation of an animal-centred perspective; (4) a focus on promotion of positive animal welfare states (not just avoidance of negative states); and (5) an exploration of ways that machines can support a reduction in the exposure of animals to negative human-animal interactions to reduce negative, and increase positive, experiences for animals.

## Introduction

Modern-day management of animals is based upon two principles, whereby management practices need to: (1) comply with the objectives of monetary profit, benefit and/or pleasure and (2) comply with humane care of animals and legislative requirements for their care (Hemsworth 2007; Acharya *et al.* 2022). Human-animal interactions (HAIs), both direct and indirect, are a key feature of animal management (Acharya *et al.* 2022), both in terms of the interactions that occur between animals and known people (e.g. owners/caretakers, animal managers, animal handlers) and unknown people (e.g. members of the public, visitors). There are additional interactions with people not completely unknown to animals, but who are less familiar (e.g. veterinarians or healthcare providers). Animals may have different interactions with these distinct groups, potentially building up strong relationships with familiar people (Patel *et al.* 2019). However, interactions between animals and people are not always positive and the valence of these may be impacted by the particular type of interaction and the animal’s perceptions of it.

It has been acknowledged that, for animals under human care, HAIs may have impacts on animal welfare and experiences, regardless of the area (e.g. in laboratories, companion animals, zoo animals, farm animals) (Hosey & Melfi 2014). But the types of interaction to which animals in each of these areas are exposed may vary widely. Understanding the impacts of HAIs on animals has ramifications in terms of animal experiences and welfare (Hosey 2000; Sherwen & Hemsworth 2019; Rault *et al.* 2020), animal handleability or response to handlers and keepers (Brajon *et al.* 2015b; Ward & Melfi 2015), animal productivity (Hemsworth *et al.* 1989, 1993) and animal health (Gross & Siegel 1982; Waiblinger *et al.* 2006).

In recent years, there has been a significant increase in the automation of processes across a range of disciplines. This has also occurred within animal management. Automation within the animal sector may include using robots to undertake tasks that replace or support people (e.g. on farms, robots are being utilised to organise animal feed, or enable cows to choose when to be milked through the use of automated milking systems; Bhoj *et al*. 2022). Automation may also work to: (1) enrich an animal’s life (e.g. in zoos there has been a drive towards creation of technological enrichment which provides cognitive challenges to the animals; Clark *et al*. 2019); (2) to support the care of animals (e.g. using technology to facilitate welfare assessment or animal management such as using cameras or wearable technology in zoos; Diana *et al.* 2021); (3) to assess physical health and animal location (e.g. on-farm; Gehlot *et al.* 2022) or to enable companion animals to enter or exit homes through doors operated by microchip readers (Sure Pet Care 2023). The development of this technology, and specifically the manner in which the animals are potentially interacting with technology, are described here as machine-animal interactions (MAIs). As with HAIs, the impact of MAIs on animals has implications for their experiences and understanding this is important in promoting good health and welfare.

This paper presents findings from a workshop involving stakeholders and researchers from the farm, companion and zoo animal disciplines, to better understand the animal welfare implications of human-animal and machine-animal interactions and their future in an increasingly technologised world.

### HAIs/MAIs on-farm

On farms, animals may perceive interactions with humans as positive, negative, or neutral (for a detailed review, see Zulkifli 2013). There is a need for a range of interactions between farm staff and animals from the purely observational, such as mobility scoring of cattle, through to physical handling and restraint for procedures such as foot trimming and artificial insemination. All of these interactions may have differing impacts on animals, and these impacts will be affected by numerous factors, including: what the interaction consists of and how the animal perceives that interaction, the housing of the animals (Fanson & Wielebnowski 2013), the previous animal-stockperson relationship (Zulkifli 2013; Rault *et al.* 2020), ontogenetic developmental factors (e.g., previous experience) (Weinberg & Levine 1980; Spiezio *et al.* 2021) and the predictability of the interaction (Weinberg & Levine 1980). Historically, research has focused upon the former factors, with less focus on the predictability of the interaction. Predictability takes two forms: temporal predictability where events occur at fixed or variable intervals, and signal predictability, which relates to the reliability of the signal for a following event (Bassett & Buchanan-Smith 2007). Animals’ perception of predictability can vary between individuals, populations, and species; it may be linked to cognitive capacities, but also to previous experiences (e.g. what the animals expect, and have previously experienced, when humans are near them or interact with them). Whilst less predictable human behaviours are often seen as more negative, and predictable human behaviours are seen as more positive, trust and familiarity may also affect the interpretation of the interaction (Destrez *et al.* 2013; Brajon *et al.* 2015a,b). Predictability is inherently linked to animal control, which has implications for animal welfare (Bassett & Buchanan-Smith 2007). It is likely that the way farm animals interpret human-animal interactions is quite nuanced (Nawroth *et al.* 2019; Jardat & Lansade 2022). The use of MAIs on farms has the potential to reduce these unpredictable interactions and thus avoid situations that may negatively impact animal welfare.

Technological advancement has perhaps been most significant within the farm animal industry. Technologies have been used on farms for thousands of years, with animals themselves initially being part of those technological advancements, as an aid for humans. For example, oxen (*Bos taurus*) were used as a substitute for manpower, to pull the plough. Nowadays, extensive technology use on-farm is enabling unprecedented process automation. For example, on dairy farms, automation takes place at the farm level (e.g. milking parlours such as rotary or automatic milking systems and video surveillance for lameness and body condition scoring monitoring), group level (e.g. heat-activated fans in the shed and feed and slurry management) and at the individual level (e.g. calving detection, oestrous, rumination, locomotion monitoring and health monitoring). The need for process automation has increased as farms have moved from being small hands-on facilities to being large intensive commercial systems, compounded by the increasing challenges around farm labour (Rutter 2012). Historically, farming was driven predominantly by production, but in more recent times, particularly with changes in public attitudes and consumer pressure, the industry has moved to a more holistic approach with consideration for animal welfare and animal cognitive abilities, which may have helped to drive this technological advancement. However, the development of animal welfare science within industry is at different stages throughout the world and its interaction with local culture differs across geographic areas (Marchant *et al.* 2023).

### HAIs/MAIs in the companion industry

Human-animal interactions with companion animals are often considered from an anthropocentric perspective. The ‘pet effect’ is defined as the mutually beneficial relationship that forms between people and their pets. There is a large body of research which has focused upon this concept, predominantly with the aim of exploring whether owning a companion animal is beneficial for human health, well-being and quality of life (Ein *et al.* 2018; Scoresby *et al.* 2021). Owner interactions with companion animals are copious and may range from caring and playing to training, undertaking working roles and conducting exercise-based activities. The types of interaction may vary in relation to the owner’s interaction style (Cimarelli *et al.* 2016), or in relation to the role of the companion animal (for example, some companion animals assume working roles, such as assistance animals, or performance roles in sports). With such a range of HAIs associated with companion animal ownership, it follows that HAIs can have a wide spectrum of effects for companion animals. Within the equine industry, the importance of understanding factors which influence owner behaviour within owner-equine interactions has been highlighted (Luna & Tadich 2019), and this is equally important in other companion animal industries too. Beyond interactions with owners, companion animals may also interact with a wider circle of humans including family members, friends, neighbours, veterinary professionals and the wider public, all of which have an impact on the animals’ experience.

As computing-enabled technology becomes ever more pervasive in human activity, technology-based interactions are becoming more likely for companion animals – from video-mediated communication that enables owners to monitor their companion animals remotely to wearable technology to track animals’ location, behaviour and health parameters (Jukan *et al.* 2018). The result is that humans, companion animals and technology are becoming increasingly entangled in a multiplicity of interactions. There is certainly scope for the use of technology to improve the welfare of companion animals. Through the provision of more information/data, improved owner awareness can be attained. For example, Nelson and Shih (2017) found that the provision of personalised and quantifiable pet data, in some instances, strengthened the human-animal bond and provided opportunities to improve animal health. However, researchers alongside owners share concerns regarding impacts technology may have on companion animals (Ramokapane *et al.* 2019). For wearable technologies in particular, concerns around the comfort of the animal must be addressed to ensure animal welfare is not compromised. For example, Paci et al. (2017) found cats (*Felis catus*) were sensitive to the design of trackers that attach to collars, expressing behavioural indicators of discomfort. Where technology is focused on remote interactions between companion animals and people, concerns have been raised about the negative effects this might end up having on animal welfare and human-animal relations, with authors suggesting technology should be used to support people’s caring practices, not replace them (van der Linden *et al.* 2022). Others have argued how it is essential that technology is designed by taking an animal-centred approach, with the goal of improving animal welfare and offering opportunities for positive experiences (Mancini 2011).

### HAIs/MAIs in the zoo industry

Within the zoo industry there are three principal groups of people that animals routinely interact with: visitors, keepers and other staff within the zoo (including maintenance staff and veterinarians). Each of these three groups of people bring different experiences to the animals, which will impact upon the building of positive, negative or neutral relationships (Patel *et al.* 2019). The level of interaction with these groups of people is also very variable. Visitors may range from passively observing animals, to attempted (solicited or unsolicited) interaction through banging on enclosure windows or otherwise trying to capture the attention of animals, or being in closer proximity through animal experiences (Sherwen & Hemsworth 2019; Spooner *et al.* 2021).

Keepers, in particular, are likely to have a physically closer relationship with the animals they routinely work with. Positive keeper-animal interactions (KAIs) can be beneficial for animals as they can lead to increased reproductive success (Mellen 1991), increased affiliative behaviours (Baker 2004; Manciocco *et al.* 2009), increased play (Manciocco *et al.* 2009) and reduced distress calls. Repeated positive interactions can lead to the development of positive relationships, with these relationships differing with different staff (Ward & Melfi 2015). Visitors within zoos typically have one of three impacts on animals, namely negative (where visitors cause negative stress to animals), neutral (where there are neither positive or negative impacts on animals), and positive (where visitors are a positive stimulant for animals) (Sherwen & Hemsworth 2019; Ward & Sherwen 2019). There are a number of factors that have been identified as predictors of animal responses to humans in zoos, including but not limited to: ecological variables (e.g. habitat type); animal size; animal rearing history; previous experiences and individual animal personality (Davey 2007; Carder & Semple 2008; Choo *et al.* 2011; Queiroz & Young 2018; Sherwen & Hemsworth 2019; Hashmi & Sullivan 2020; Spiezio *et al.* 2021; Hosey *et al.* 2023).

Within zoos there is a suite of ways which technology has been used, including to enhance the visitor experience, facilitating non-invasive measures of animal behaviour and creating more cognitively advanced environments for animals (Clay *et al.* 2011). It is also extensively used in wildlife conservation (Pacheco 2018) and is being increasingly used to monitor animals, particularly in relation to the use of artificial intelligence to assess welfare (Congdon *et al.* 2022). As discussed, it is important that technology enhances rather than prevents the strong positive bonds that are capable of forming between stockpeople and the animals they work with. Technology that has been used so far within zoos has principally focused upon enriching animals’ experience, particularly in terms of provision of cognitive enrichment opportunities (e.g. touch screen tasks; Egelkamp & Ross 2019), or to support routine animal husbandry (e.g. automated feeders; Haspeslagh *et al.* 2011).

### Workshop aims

The aim of this work was to bring together stakeholders and researchers from the farm, companion and zoo animal disciplines, to discuss the role of human-animal interactions and machine-animal interactions across these managed environments, foster collaborative approaches to better understand the animal welfare implications of human-animal and machine-animal interactions and to consider the future of machine-animal interactions for animals under human care, with particular considerations towards the animal welfare implications of these interactions.

## Materials and methods

### Study design

Participants from a variety of disciplines attended the Animal Welfare Research Network workshop on human-animal and machine-animal interactions at Harper Adams University, UK on 26 April 2023. The workshop consisted of 30-min plenary talks from four research specialists in the relevant areas (farm, zoo and companion animals). It then ended with round-table discussions, with questions provided to participants to be used as a framework for guiding discussions. An outline of the workshop programme is provided in the Supplementary material. The purpose of the plenary talks was to provide a background to the workshop, introducing the audience to HAIs and MAIs in disciplines which may be outside of the audience’s own focus, and to provide stimulation for fruitful discussions in the afternoon.

The plenary talks focused on:

Talk 1: Predicting and interpreting animal behaviour, taking a cognitive approach to our interactions with animals, both in terms of human-animal and machine-animal interactions, particularly in relation to farm animals;

Talk 2: Human-animal interactions in zoos and the potential welfare implications of those interactions, the ways in which robots are currently used in zoos and some of the potential impacts of those;

Talk 3: The use of technology within the farming industry, including the pros and cons of that technology; and

Talk 4: Animal-computer interactions (with a particular focus on companion animals) and the impacts technology has on animal welfare and human-animal interactions.

In total, there were 38 delegates, all from Europe, with the majority being UK-based. They were from a range of disciplines (farming; n = 16, companion and equine; n = 10, zoo; n = 2, mixed discipline; n = 3, non-species specific; n = 3, and four which were unknown). Delegates were split over five mixed-discipline focus groups. Group members were assigned according to known research background as far as possible to ensure an even representation across the disciplines. The groups were each given 75 min for the focus group activity. As a general guide for semi-structured discussion, participants were given five questions pertaining to human-animal and machine-animal interactions and were asked to identify three priority areas for future research, with some questions included to facilitate initial discussions. The questions for discussion were as follows:

- How did the HAIs described by the speakers differ from your industry/your experiences?
- Do you think the opportunities and types of human-animal interactions and machine-animal interactions differ across industries?
- Did anything in particular stand out to you from the talks?
- How might we build on some of the things discussed in the talks?
- What should our research priorities be? Both within and across disciplines, how can we learn from one another?

A designated notetaker from each focus group was asked to document the key discussion points and priority research areas. Participation was optional at the round-table discussions. All participants at the workshop gave written consent for the information they provided during discussions to be collated and shared via publication in an anonymised format. At the end of the focus group, the three priority research areas from each group were presented to the audience by a member of each group. These were audio-recorded (with participant consent) and transcribed. Ethical approval was granted via the Harper Adams University Ethics Committee (project approval number 0123-202302-STAFF). All information relating to the identity of participants or places of work was removed prior to analysis.

Informal analysis to capture key discussion points from the day was undertaken based on the written notes taken by the working groups. The notes served to answer questions on the benefits and challenges of HAIs and MAIs for animals under human care, including examples participants gave in relation to those areas. A thematic analysis was then undertaken to identify the three research priorities arising from this workshop, based upon the transcripts from the groups’ presentations back to the audience. The ‘central organising concept’ (Joy *et al.* 2023; p 156) was the triad of human-animal-technology interactions (Hirsch-Matsioulas & Zamansky 2020), with generated themes representing priority research areas to optimise animal and human welfare within these interactions. As previously stated, participants originated from a variety of industries, with research and practical backgrounds relating to farm, companion, zoo, wildlife, and laboratory animal welfare. Thus, participant responses were likely informed by multiple conceptual frameworks of animal welfare and a predominantly inductive approach was employed in the development of themes. Themes were mainly developed early on, with some recursive evolution throughout the process. Following Braun and Clark (2023), a codebook approach was selected, with NVivo (v 1.7.1, QSR International, Cheshire, UK) used to facilitate coding.

## Results and Discussion

### Results of the informal analysis: Capturing key discussion points

#### What are the benefits, challenges or limitations of human-animal and machine-animal interactions for animals under human care?

An overview of the more broadly considered benefits and challenges/limitations of machine animal interactions within the companion, farm and zoo animal industries identified by the participants, is detailed in Table 1.

**Table 1.** An overview of the benefits and challenges/limitations of machine-animal interactions for animals under human care as reported by workshop delegates. Additional points have been added in by the authors to further explain some of the reported comments. Quotes taken from the written notes made during the workshop are represented with single quotation marks. These have been used to provide examples of the points raised by participants

Benefits identified by workshop participants
Reduction of negative human–animal interactions (e.g. those which are potentially dangerous to humans and animals or cause conflicts), which gives scope for the remaining HAIs to be more positive for both humans and animals
Greater opportunity for animal choice/agency e.g. ‘Good for agency as animals get to choose milking’
Adding value to the animal
**Challenges/limitations identified by workshop participants**
In some industries animals are with humans for a shorter period of time which reduces familiarity with humans, this can then have consequences when humans are trying to work with the animals e.g. ‘Pig and poultry fast turnaround so less interaction with humans so harder to move them in abattoir’
Money/cost e.g. ‘Technology is expensive so farmers may have to have more cows to pay for them – this could then lead to higher stocking densities and potentially reduced welfare.’
Pressure to discard what is most appropriate for the animals e.g. ‘Huge pressure to discount view of animals because other things speak louder’ ‘An animal–centred approach is necessary … but is not always used.’
Lack of knowledge/public education and a lack of information or misinformation
Potential for technologies to go wrong or break e.g. ‘Technology is a tool but it can go wrong’, ‘Technology not doing what it should be doing’
Reduced opportunity for interactions with animals or reduced time spent with animals e.g. ‘If we replace all interactions then we may not see animals as much’
Potential barriers to uptake – but it is not necessarily known what those are
There may be a disconnect between the outcome and the animal
Technology could have negative impacts on the animal – e.g., when used as part of tracking ‘With wildlife, the use of technologies can often hamper reproductive success and even sometimes survival. There is a [sic] unwritten rule that the weight of the device should be no more than 5% of the body weight of the animal.’
Potential negative impacts when animals do need to interact with humans, if interactions have been reduced e.g. ‘If we increase machine interactions and reduce human–animal interactions, then it could have a negative impact when humans do need to be involved.’
Technology may be available but not always accessible, e.g. ‘AI technology available but needs lots of coding’

Although the types of machines we currently use within these areas are very different, with some robots being used to do ‘jobs’ and others as sources of enrichment for animals or for training animals to undertake particular tasks (Mancini 2023; Nawroth 2023; Neary 2023; Ward 2023), the informal discussions that were facilitated during this workshop highlighted commonalities across the three discipline areas in relation to the benefits and challenges of MAIs. This is an important point to recognise as it provides further evidence for the need for collaborative approaches to animal welfare science. This is a key aim of the Animal Welfare Research Network: “The AWRN aims to bring together the UK animal welfare research community including researchers in related areas and stakeholders with interests in animal welfare, to identify important research topics, increase collaboration, and support and encourage further research” (AWRN 2024). Whilst it is known that unique dyads may form between caregivers and animals (Ward & Melfi 2015) and that positive HAIs can bring intrinsic rewards to animals improving their experiences (Rault *et al.* 2020), regardless of the industry, it was not known whether the same cross-disciplinary similarities would be seen when considering the use of technology within animal industries.

There has been substantial development in technological advancement in animal management in recent years, in terms of artificial intelligence, machine learning and animal-centric designs (Wolfert *et al.* 2017; Neethirajan 2020; Webber *et al.* 2022). Whilst this rapid development drives innovation and moves the industry forwards, there are challenges which come with it. These were highlighted by workshop participants. Across all industries, there were more challenges and limitations identified than there were benefits. Whilst there were fewer benefits in number these were consequential with the potential for MAIs to have a big positive impact on animals, including provision of choice and enablement of agency, and adding value to the animal as an individual. It is clear from these discussions that this is an area which requires deeper consideration as it has significant potential benefit to animal welfare. Many of the challenges identified during this workshop can be overcome with further research, particularly as regards cost, or apprehension concerning the implementation of technology. For example, concerns around cost could be overcome by undertaking research to improve the effectiveness and efficiency of technologies. Efficiency could be improved by using machine learning to process large amounts of data to provide highly detailed information about the behaviours and states of monitored animals, in some cases using ambient sensors rather than instrumenting individual animals (Hansen *et al.* 2018; Anagnostopoulos *et al.* 2023; Siachos *et al.* 2023). This means that once the technology is in place, monitoring an increasing number of animals does not necessarily increase costs exponentially. The research priorities detailed below provide specific priorities which the stakeholders involved in this workshop believe are important for advancing this scientific field.

#### What are the broad areas for consideration/what should we be doing to advance this scientific field?

Areas for consideration and related actions to be aimed for were roughly grouped into fourteen categories (Table 2). They principally entailed considerations into the design of technology (making sure it is animal-centred and evidence-based), multidisciplinary collaboration, communication and knowledge dissemination, knowledge acquisition, and consideration of different perspectives (e.g. animals, humans). Examples of comments made in relation to those categories are provided in Table 2.

**Table 2.** An overview of the examples that were given by participants in relation to those key areas identified by stakeholders as important when considering advancement of science in HAIs and MAIs for animals under human care

Category	Examples
Application (n = 19)	Supplement animals with technology rather than replace Use tech to improve interactions with animals Using AI to measure positive welfare How will tech work within a system? What drives the tech? What is the incentive? Consent in animals – what does it look like? Complexities of reality Options for agency/positive reinforcement Who is the tech designed for? What is it designed to do?
Gaining knowledge (n = 15)	Interpretation of animal behaviour and how animals learn What do different industries need? What are the barriers to uptake? How do we overcome these? Understanding animal perceptions/reactions – identifying what’s important to them within the HAI and understanding what they need in terms of MAIs Recognising individual animal differences (e.g. personality, impacts of age, life cycle etc) Recognising species sensory perceptions Understanding how to make tech adaptable to all facilities (not all facilities are the same) How to assess/promote welfare How tech affects HAIs What sounds will be produced by the tech Consideration of one animal vs multi animal groups Understanding what the outcomes mean in terms of interaction with the tech (are they positive or negative)
Collaboration (n = 9)	Collaboration between people who are designing products for stakeholders and animal behaviour and welfare specialists Consider a worldwide perspective Identify ways to share information across disciplines Combining tech solutions to increase accuracy Interdisciplinary research Cross sector learning Communication
Animal–centred design (n = 7)	Recognise and encourage animal–centred design Animals need [to experience] agency, equal opportunities and opportunities for control over their environment How to design systems using animal–centred approaches Animals as stakeholders
Education (n = 6)	Public awareness Education for stakeholders Embedding continuing professional development
Evidence–based design (n = 4)	Animal behaviour/welfare is supporting decisions/development of technology Scientific evidence Responsible innovation frameworks Checklists/guidelines for tech development
Knowledge dissemination (n = 2)	Reduce publication bias Disseminate results
Cost (n=2)	Where is the money?
Other species (n = 2)	Poultry Insects Cameras and machine learning across all species
Different perspectives (n = 2)	Individual animal and human traits What is the impact of animals being kept for different purposes (e.g. for conservation or breeding vs domestics)
Public perception (n = 1)	Understanding public perception
Validation (n = 1)	Validation is needed
Legislation (n = 1)	Should there be more legislation?

Participants engaged in informal discussions about research priorities, before providing their top three recommendations. Some of the points included within the informal discussions covered more general areas (e.g. science communication). These are included in this report partially for completeness, as a true reflection of what was discussed during the day; but they also, importantly, show the relevance of cross-disciplinary work such as this, indicating that wider issues remain relevant in all disciplines in relation to animal welfare. Thus, moving forwards as a field, we should be facilitating cross-disciplinary communications to maximise knowledge sharing and learning from experience. The workshop was designed to promote reflection on the impact of technology (both positive and negative) on HAIs and MAIs and the participants’ experience of these fed into their discussions. Perhaps unsurprisingly, due to the newness of this field, this reflection prompted participants to ask questions to which they do not have answers. Indeed, many of the points covered in Table 2 included questions (e.g. what does consent in animals look like?). A key challenge that has been identified in animal-centric design approaches is understanding how animals can tell us what they need or want (North & Mancini 2016; Mancini 2017; Webber *et al.* 2022). The development of science relies upon reflection and probing and so this workshop, which enabled critical discussion, is highly important for the development of the field.

### Results of the thematic analysis: Identification of research priorities

#### Bridging the gap between different industries through interdisciplinary collaboration

The most common theme (raised seven times across the five presentations) centred upon interdisciplinary collaboration. Specifically, participants discussed the need to bridge disciplinary gaps between different industries. This research priority was well encapsulated by Groups C and E:

*“The need for more multi- or interdisciplinary-driven work, so that you have people in the room who can offer some common sense through viewing the same challenge through different lenses”* [Group C].

*“…can we do cross-sector learning? What has already been done but we don’t know about in farming because it has happened in zoos, but actually if we just make a few tweaks readily translates to a different setting?”* [Group C].

*“…looking at a multi-species, machine-vision approach for monitoring all of this… you could definitely put a lot of stuff that we see in farm into zoo and vice versa”* [Group E].

These research priorities align with observations made by Jukan *et al*. (2018; p 19) following a comprehensive literature review: “We have found a lot of common features in how the animal-based sensor network systems are built and used, but little or no evidence that the systems can be reused across species or animal applications.” Thus, as explained by Group A, the lack of integration of cross-sectoral research is a barrier that can only be overcome through collaboration between key stakeholders, animal behaviour and welfare specialists, academics, and funding bodies. Without this collaboration, research efforts risk becoming futile, as described by Groups B and D:

*“Overarching everything is the need to include other disciplines around human behaviour change, for example. So it’s all very well developing this ideal kit but if… farmers* [and other stakeholders] *are unwilling to use it then that’s an issue”* [Group D].

*“Actually identifying what the different industries need… acknowledg*[ing] *that not all facilities are the same… How do we recognise that differentiation and support that differentiation?”* [Group B].

*Ensuring that developed technologies undergo rigorous validation studies and are subject to quality assurance protocols and respecting animal-centred design principles*

Participants also addressed other barriers to the uptake of animal-welfare-focused technologies, including the necessity for validation and quality assurance:

*“Lots of companies are producing these technologies with certain claims they do X, Y and Z, but there is a real need… for basic applied research to validate this technology in a range of different formats”* [Group D].

*“Understanding barriers of developing quality assurance – if you are going to have the tech you need to have either legislation around it or some sort of third-party quality assurance”* [Group E].

“…*there might be a framework or checklist that we could develop where if you are developing tech these are… the steps you should probably take… to get a standard approach for developing these sets of tools to try and avoid problems like* [Speaker D] *talked about with the calving sensor”* [Group C].

Group C’s insights were prompted by a discussion that took place earlier in the Workshop regarding potential animal welfare risks of digital technology. For instance, tail-mounted sensors can be used to detect the onset of calving, ensuring calving assistance, if necessary and thus reducing the risk of stillbirths. However, studies have found that such devices can cause pain and swelling on the tail, and therefore must be removed (Voß et al. 2021). As Mancini *et al*. (2017; p 130) explains, “the possibility of designing *for* animals, let alone *with* animals’ faces fundamental obstacles including interspecies communication barriers and misalignment of human and animal interests”. Workshop participants also expressed uncertainty as to how designing *with* animals, or animal-centred design (ACD), could be achieved in practice, particularly in industries in which it has not been a historic focus: “*How do you actually design a trial or system or a technology by using an animal-centric approach and what does getting animal consent mean or look like, and how do you achieve that in practice?”* [Group C].

On the other hand, other groups highlighted the importance of ACD, coupled with recommendations, including targeted education and consideration for the individual animal as a future research imperative:

*“Make sure animal-centred design is at the forefront… Education about animal behaviour and learning for every stakeholder at every level* [is needed]” [Group A].

*“….typically in a research question we are often studying at a group level looking for our scientific outcomes. But actually, don’t lose sight of individual variation in responses of animals… there’s a piece of research to do in there around better understanding individual differences in animals”* [Group D].

Mancini (2017) highlights the importance of enabling animals to inform the process of design of technology, through an iterative process of incremental orientation towards an optimal outcome that might never be fully achieved but that can nevertheless be approximated. During the process, designs are negotiated and evolve through ongoing interactions both between humans and animals directly and via technology. In this regard, animal-centred research set-ups and the kind of interactions they foster are key to lead to design outcomes that are beneficial for animals and human stakeholders (Mancini & Lehtonen 2018).

However, workshop participants also echoed concerns raised by Bos *et al*. (2018; p 85) over the potential for technology to disregard animals’ “individual qualitative differences”. Technology exists to measure individual animal behaviour and improve our understanding of individual animal variation, but this technology has often been developed with other primary goals in mind, such as reduction of disease incidence (Beaver *et al.* 2020). The focus on minimising negative welfare states, rather than promote positive welfare states, still predominates in certain industries; for instance, Schillings *et al*. (2021) argue that the current ability of Precision Livestock Farming (PLF) technologies to promote positive welfare continues to be somewhat limited. At the same time, in other industries, much of the technological development has focused upon improving assessment of, rather than promoting, animal welfare, although the need for further research is still recognised (Whitham & Miller 2016; Diana *et al.* 2021).

### Technologies to promote positive animal welfare and exploring the ways in which technology may mediate human-animal interactions

Group B advocated a move towards developing more technology to promote positive animal welfare, specifically the use of technology as enrichment. This group also invoked the One Welfare principle of a bidirectional relationship between human and animal welfare (Pinillos *et al.* 2016), calling for research into technology that could mediate that relationship, e.g. “*Identifying technology which can make the staff/stockperson welfare better, because that then leads into positive human-animal interactions as well”* [Group B]. For example, robotic milking can improve cow welfare by allowing the cows to choose when they are milked (Jacobs & Siegford 2012) whilst also reducing labour demands on farmers (Rodenburg 2017), leaving more time for positive HAIs.

Participants further suggested researching the valence of specific elements of human-animal interactions to identify which specific aspects might lend themselves to replacement, in order to focus upon replacing aspects which might lead to experiences of negative valence whilst providing extra opportunities for time to be spent engaging in positive HAIs; *“Trying to understand what is important to animals within* [specific] *human-animal interactions and what bits do we need to retain as human-animal interactions and what bits can we transfer over to an automated interaction?”*

As suggested by Bos *et al*. (2018), smart technologies have the potential to redefine the notion of care. In this regard, a judicious approach is needed to evaluate the reorganisation of human and animal responsibilities, and to decide which facets of HAIs can ethically be replaced by automated interaction.

In summary, the research priorities identified by the workshop participants centred on the following:

#### Bridging the gap between different industries through interdisciplinary collaboration

It is only through the inclusion of all relevant stakeholders, including the animals, and targeted education that differences in facilities can be supported and technology can be designed that will be used in practice. This interdisciplinary collaboration should be encouraged both across industries and also within academia and funding bodies. Within this it is important to recognise that not all facilities and industries will be the same, and so consideration should be given as to how to make the technology fit for purpose and how to overcome industry or individual site-specific barriers to uptake.

#### Ensuring that developed technologies undergo rigorous validation studies and are subject to quality assurance protocols

There are many potential risks associated with using technology for animals under human care. Malfunctions could lead to reduced welfare for animals. A lack of validation may lead to a reduced uptake of beneficial technology. Considering development of quality assurance protocols will reduce the potentially negative impacts of technology.

#### Respecting animal-centred design principles

And, relatedly, considering individual animal experiences, responses and behaviour. It is important to recognise animals as individuals and provide them with opportunities to exhibit control or choice within their environments, thus utilising technology in a way which enhances welfare at an individual animal level. We should aim to utilise the technology to help to understand the animal’s reaction, recognising animals as individuals and paying particular consideration to their sensory perceptions, understanding the impact of the technology from the animal’s point of view.

#### Focusing on technologies to promote positive animal welfare

Also including technologies used for enrichment. Development of technologies which promote positive animal welfare will help to develop environments in which animals under human care can thrive, not just survive. Education will help to facilitate positive animal welfare across disciplines.

#### Further exploring the ways in which technology may mediate human-animal interactions

With particular emphasis on reciprocal improvements between animal and human welfare. Investigation is needed into negatively valanced HAI elements that are replaceable by technology, and also considering how the technology can be used to make staff/stockperson welfare better, which will then lead to more positive HAIs.

### Animal welfare implications

Human-animal interactions are intrinsically linked to animal welfare, with a range of factors affecting the animal’s perception of the interaction. These factors relate both to the interaction itself (e.g. to the form of the interaction and its predictability) and to the animals themselves (e.g. animal personality or individual experiences having implications for animals’ perceptions). The advancement of any scientific discipline, but particularly in this very applied area, is most successful through collaborative efforts, with knowledge sharing being an important aspect of evidence-based management of animals under human care. Accordingly, in this research we utilised an opportunity offered by the Animal Welfare Research Network to host an interdisciplinary workshop. The output of that workshop and this resulting work is a series of research priorities in relation to HAIs and MAIs for animals under human care, which will be of benefit to scientists and animal carers who are working to ensure positive animal welfare for animals as the field of automation continues to advance.

## Conclusion

Human-animal interactions vary across animal industries and across individual experiences, with the nature of the interaction being affected by a suite of different factors that may be beyond the control of the individual animal. Although there are numerous potential benefits of utilising technology within animal industries, it should not be assumed that technology use necessarily equates to improvements in animal welfare. It is important that any technologies that are used to inform knowledge of animal welfare states are validated and clearly identified as fit for purpose. The positives and negatives of technology must be considered within industry, but technology cannot and should not replace good animal husbandry; rather, it should work as an aide to support practitioners, streamline processes and improve animal welfare. Based on the results of this workshop, it is recommended that application of machine-animal interactions within animal industries focuses on interdisciplinary collaboration, the incorporation of animal-centred design, the promotion of positive animal welfare states (not just avoidance of negative states), and an exploration of ways that machines can be used to support a reduction in the exposure of animals to negative HAIs.

## Supporting information

Williams et al. supplementary material Williams et al. supplementary material

## Supplementary material

The supplementary material for this article can be found at https://doi.org/10.1017/awf.2024.23.
