# Supplementary material for: Human-animal interactions and machine-animal interactions in animals under human care: A summary of stakeholder and researcher perceptions and future directions
Source: Anim Welf. 2024 May 9;33:e27. doi: 10.1017/awf.2024.23 (PMC11094549; doi:10.1017/awf.2024.23)
Supplement: Williams et al. supplementary material [file S096272862400023Xsup001.pdf]

Animal Welfare Research Network (AWRN) Programme

09:30 – 10:15: Registration (with tea and coffee)

10:15 – 10:25: Workshop overview

10:25 – 10:35: Welcome from Harper Adams University Vice Chancellor, Professor Ken Sloan

1035: – 12:35: Invited speakers (30 mins talk, 10 mins questions)

- 10:35 – 11:15: Dr Christian Nawroth
- 11:15 – 11:55: Dr Samantha Ward
- 11:55 – 12:35: Professor Clara Mancini

12:35 – 13:25: Lunch and networking

13:25 – 14:05: Invited speakers (30 mins talk, 10 mins questions)

- 13:25 – 14:05: Dr Joseph Neary

14:05 – 15:20: Workshops (round table discussions)

15:20 – 15:45: Tea, coffee and networking

15:45 – 16:15: Reports from workshops

16:15 – 16:20: Conference close
